# Prenatal diagnosis of critical congenital heart disease associated with lower postpartum depressive symptoms: a case-control study

**DOI:** 10.1016/j.jped.2024.06.011

**Published:** 2024-08-09

**Authors:** Daniela R. Vieira, Patrícia P. Ruschel, Márcia M. Schmidt, Paulo Zielinsky

**Affiliations:** a Instituto de Cardiologia do Rio Grande do Sul- Fundação Universitária de Cardiologia (IC-FUC), Programa de Pós-Graduação em Ciências da Saúde, Cardiologia, Porto Alegre, RS, Brazil; b Instituto de Cardiologia do Rio Grande do Sul- Fundação Universitária de Cardiologia (IC-FUC), Serviço de Psicologia Clínica, Porto Alegre, RS, Brazil; c Instituto de Cardiologia do Rio Grande do Sul- Fundação Universitária de Cardiologia (IC-FUC), Unidade de Cardiologia Fetal, Porto Alegre, RS, Brazil; d Universidade Federal do Rio Grande do Sul (UFRGS), Departamento de Pediatria, Porto Alegre, RS, Brazil

**Keywords:** Congenital heart disease, Postpartum depression, Pregnancy, Fetal diagnosis

## Abstract

**Objective:**

When the expectant mother is faced with an unforeseen event during pregnancy, she may experience emotional fragility and depression. This study was carried out to test the association between the time of diagnosis of critical congenital heart disease (CCHD) and depressive symptoms in puerperal women.

**Method:**

A case-control study. All mothers answered a semi-structured questionnaire and the Edinburgh Postnatal Depression Scale (EPDS). Pearson's correlation and multiple linear regression analysis were used to determine factors associated with depression.

**Results:**

50 puerperal women, 23 cases and 27 controls. The proportion of puerperal depressive symptoms was 26.1 % among mothers of infants prenatally diagnosed with CCHD and 77.8 % among mothers of infants postnatally diagnosed (*p* = 0.001 [OR] 9.917; 95 % CI 2.703–36.379). Multiple linear regression analysis showed that the use of psychotropic drugs and time of diagnosis were significantly associated with puerperal depressive symptoms.

**Conclusion:**

Prenatal diagnosis of CCHD was associated with significantly lower levels of depressive symptoms.

## Introduction

Pregnancy brings about to the expectant mother emotions, idealizations, and expectations of an uneventful pregnancy and a healthy baby.^1^ For years pregnancy was regarded as a quality, peaceful time. Yet the expectant mother must adapt herself both physically and emotionally to the changes of pregnancy and puerperium and this experience may exacerbate psychiatric symptoms or disorders.^2^ Adaptive coping skills and personality traits play a role in the adaptation to changes but these are times of increased vulnerability for emotional disorders such as depression.^3^ When parents learn that their baby has a birth defect they respond in various ways and experience different feelings resulting from the pregnancy idealization, cultural values, support network, and the couple's maturity and beliefs.^4^

Fetal echocardiography is an essential tool for intrauterine diagnosis and evaluation of fetal heart for structural or functional alterations beginning at 18 weeks of gestation.^5^ Time of diagnosis is key for childbirth planning and addressing puerperal depressive symptoms. Early diagnosis and appropriate medical intervention during pregnancy can improve perinatal outcomes and prevent considerable postpartum emotional distress as well as help in planning medical and/or surgical interventions after birth.^6^^,^^7^

A study comparing the psychological functioning of parents of infants prenatally and postnatally diagnosed with congenital heart disease (CHD) found that, regardless of the time of diagnosis, parents of newborns with severe CHD experienced high levels of psychological distress, including depression.^8^ In contrast, a prospective cohort study with parents of children prenatally diagnosed with complex CHD supported the perception that prenatal diagnosis has a positive impact on postpartum emotional symptoms. Pinto et al,^9^ examined the association of the time of diagnosis (prenatal or postnatal) with symptoms of stress. They found that the levels of symptoms of stress were significantly lower when parents learned about the diagnosis prenatally. This study also showed that mothers experienced higher levels of anxiety and stress than fathers.

Given the importance of this subject and the scarcity of studies in the literature, the present study aimed to examine the impact of the timing of CHD diagnosis on the levels of depressive symptoms in mothers of infants with prenatal vs. postnatal diagnosis of this condition.

## Methods

### *Study location and period*

This study was carried out at a tertiary referral center for cardiac care in southern Brazil in two waves: from December 2019 to March 2020 (temporarily interrupted at the onset of the COVID-19 pandemic) and then from August 2021 to March 2022.

### *Study design*

Case-control study. Puerperal women with depressive symptoms were inquired about the time they learned about the diagnosis of their infants. Cases were puerperal women of infants prenatally diagnosed with CCHD by fetal echocardiography. The control group consisted of puerperal women of infants postnatally diagnosed with CCHD. The authors examined the association between the time of diagnosis and levels of puerperal depressive symptoms in mothers of infants with CCHD.

### *Study participants*

The study sample included puerperal women aged 18 years or more of infants diagnosed with the following types of CCHD: 1) atrioventricular canal defects – severe right ventricular outflow tract obstruction, including pulmonary stenosis and pulmonary atresia; 2) severe left ventricular outflow tract obstruction, including critical aortic stenosis, hypoplastic left heart syndrome and obstructive lesions of the aortic arch; 3) congenital heart defects with parallel systemic and pulmonary circulation, including transposition of the great vessels; and 4) heart defects with anomalous pulmonary venous return, including total anomalous pulmonary venous return. Postpartum women unable or unwilling to respond to questions at the time of the interview were excluded. All women signed a free informed consent form agreeing to participate.

### *Assessment instrument*

The Edinburgh Postnatal Depression Scale (EPDS) is a 10-item questionnaire to screen postpartum women for classic symptoms of depression, including feeling guilty, sleep disturbances, low energy levels, anhedonia, and suicidal ideation. The final score is the sum of the scores for each question (from 0 to 3 according to the reported intensity of a depressive symptom). The total score ranges from 0 to 30; a total score equal to or greater than 12 indicates a risk of depression.^10^

### *Study procedures*

Puerperal women were invited to participate in the study during their infant's hospitalization for surgical intervention. They were first asked to sign an informed consent and then were administered the EPDS. The authors used a sociodemographic questionnaire to collect information on the reasons for the child's care at the study center and the time of diagnosis of CCHD by echocardiography. The EPDS was administered to all mothers before surgery, without exceptions, and there have been no refusals.

### *Variable definitions*

The authors collected information on several risk factors, including gestational diabetes, hypertension and smoking. Gestational diabetes was defined when at least two of four plasma glucose measures were abnormal during the 3-hour oral glucose tolerance test (OGTT), or fasting glucose ≥ 95 mg/dL; 1-hour blood glucose ≥ 180 mg/dL; 2-hour blood glucose ≥ 155 mg/dL; and 3-hour blood glucose ≥ 140 mg/dL.^11^ Hypertension was defined as systolic blood pressure of more than 130 mmHg and diastolic blood pressure of more than 80 mmHg.^12^ Smoking was considered present when the respondent reported smoking in the last year. The authors also collected information on prior psychiatric or psychological care and the use of psychotropic drugs. Continuous care was defined as receiving care for 2 months or more. The use of psychotropic drugs was defined as the use of the following drug classes for 1 year or more: anxiolytics, antidepressants and mood stabilizers.

### *Sample size calculation and data analysis*

For the calculation of the sample size, considering an estimated proportion of 40% vs. 70 % of depression symptoms in mothers of infants with a prenatal and postnatal diagnosis of CHD,^13^ the authors calculated a sample of 42 puerperal women in each group (cases and controls), with a power of 80 % and an alpha of 0.05. Data were entered into a Microsoft Excel spreadsheet and later analyzed in SPSS version 25.0. To examine an association of demographic characteristics with the outcome in both groups, the authors performed Fisher's exact test. the chi-square test for categorical variables, and Student's *t*-test for quantitative variables. The authors carried out these same tests to compare participants with and without depression as assessed by EPDS scores. Pearson's correlation and multiple linear regression analysis were used to determine variables associated with depression based on EPDS scores. The authors performed the Kolmogorov-Smirnov test to test the normality of scores. The authors calculated the variance inflation factor (VIF) to measure the degree of collinearity of the variables; all were < 2.

### *Ethical considerations*

This study was approved by the research ethics committee of the Institute of Cardiology of Rio Grande do Sul/University Foundation of Cardiology (IC/FUC) (protocol number 26943119.9.0000.5333).

Written informed consent to participate in the study was obtained from each participant prior to data collection and written informed consent to publish study results was obtained from each participant prior to data collection.

All data generated or analyzed during this study are included in their entirety in this published article itself. Ethics approval, participant permissions, and all other relevant approvals were granted for this data sharing.

## Results

**Figure 1:**
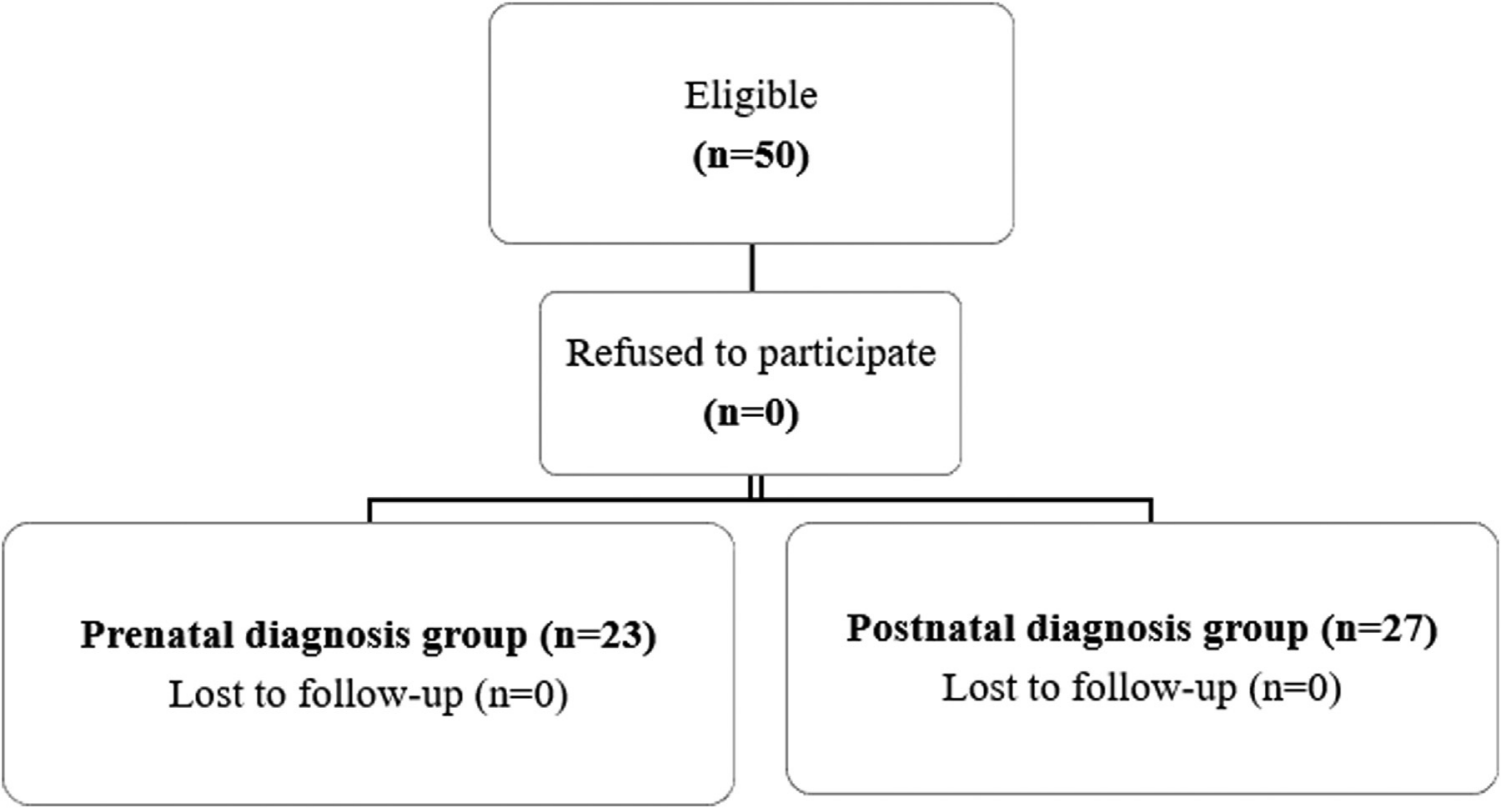
Flowchart of data collection process.

A total of 50 infants diagnosed with CCHD were hospitalized from December 2019 to March 2020 and from August 2021 to March 2022 as shown in the study flowchart (Figure 1). The authors collected data in two waves because of disruption due to the onset of the COVID-19 pandemic from 21 puerperal women during the first wave and from 29 women during the second wave. All 50 puerperal women agreed to participate in the study and none was excluded.

A large proportion of the participants lived in inner state areas (68 %) with a partner (68 %) and had an occupation (72 %). Most cases had a college education (73.9 %) and most controls had a high school education (51.9 %). All participants attended prenatal care and 62 % received care through the public system (SUS) (39.1 % among cases and 81.5 % among controls; *p* = 0.003). Age and occupation differed significantly between the groups studied (Table 1). Cases were more likely to receive prior psychiatric care than controls, but the use of psychotropic drugs was similar in both groups. EPDS scores were lower in cases than in controls.

**Table 1:** Sociodemographic characteristics and risk factors of the study participants.

**Variables**	**Total (*n*=50)**	**Prenatal diagnosis group (*n*=23)**	**Postnatal diagnosis group (*n*=27)**	p-value*
**Area of residence**				
Inner state areas	34 (68 %)	10 (43.5 %)	9 (33.3 %)	0.563
**Occupation**	36 (72 %)	21 (91.3 %)	15 (55.6 %)	0.017
**Marital status**				
Having a partner	34 (68 %)	19 (82.6 %)	15 (55.6 %)	0.067
**Age**	29.7 ± 6.2	32.6 ± 5.3	27.2 ± 5.9	<0.001
**Schooling**				
Elementary school education	9 (18.0 %)	0 (-)	9 (33.3 %)	
High school education	20 (40.0 %)	6 (26.1 %)	14 (51.9 %)	<0.001
College education	21 (42.0 %)	17 (73.9 %)	4 (14.8 %)	
**Pregnancy**				
Prenatal care at the public health system	31 (62 %)	9 (39.1 %)	22 (81.5 %)	0.003
Planned pregnancy	31 (62.0 %)	14 (60.9 %)	12 (44.4 %)	0.272
Primipara	19 (38.0 %)	10 (43.5 %)	9 (33.3 %)	0.563
Male infant	27 (54.0 %)	10 (43.5 %)	17 (63.0 %)	0.255
Participating father	45 (90.0 %)	23 (100.0 %)	22 (81.5 %)	0.054
**Risk factors**				
Gestational diabetes	11 (22.0 %)	3 (13.0 %)	8 (29.6 %)	0.189
Hypertension	8 (16.0 %)	2 (8.7 %)	6 (22.2 %)	0.261
Smoking	3 (6.0 %)	0	3 (11.1 %)	0.240
**Psychological background**				
Prior psychological care	25 (50.0 %)	13 (56.5 %)	12 (44.4 %)	0.571
Prior psychiatric care	11 (22.0 %)	9 (39.1 %)	2 (7.4 %)	0.014
Use of psychotropic drugs	8 (16.0 %)	4 (17.4 %)	4 (14.8 %)	1.000
EPDS scores	12.3 ± 6.6	10.1 ± 5.8	14.2 ± 6.7	0.024
Depressive symptoms	27 (54.0 %)	6 (26.1 %)	21 (77.8 %)	<0.001

Postpartum depressive symptoms were reported in 26.1 % of the group of prenatal diagnosis, and 77.8 % of the group of postnatal diagnosis. A comparison of these groups showed a significant difference (*p* = 0.001) (OR 9.917; 95 % CI 2.703–36.379).

Factors associated with postpartum depressive symptoms were age, schooling, time of diagnosis, gestational diabetes, and use of psychotropic drugs (Table 2).

**Table 2:** Factors associated with postpartum depression.

**Variables**	**Total (*n*=50)**	**No depressive symptoms (*n*=23)**	**Depressive symptoms (*n*=27)**	p-value*
**Age**	29.7 ± 6.2	31.7 ± 5.4	28.0 ± 6.4	0.032
**Schooling**				
Elementary School education	9 (18.0 %)	2 (8.7 %)	7 (25.9 %)	
High School education	20 (40.0 %)	20 (40.0 %)	6 (26.1 %)	0.008
College education	21 (42.0 %)	15 (65.2 %)	6 (22.2 %)	
**Pregnancy**				
Prenatal care at the public health system	23 (46.0 %)	17 (73.9 %)	6 (22.2 %)	<0.001
Planned pregnancy	26 (52.0 %)	12 (52.2 %)	14 (51.9 %)	1.000
Primipara	19 (38.0 %)	7 (30.4 %)	12 (44.4 %)	0.309
Participating father	45 (90.0 %)	22 (95.7 %)	23 (85.2 %)	0.357
**Risk Factor**				
Gestational diabetes	11 (22.0 %)	2 (8.7 %)	9 (33.3 %)	0.046
Hypertension	8 (16.0 %)	4 (17.4 %)	4 (14.8 %)	1.000
Smoking	3 (6.0 %)	0	3 (11.1 %)	0.240
**Psychological background**				
Prior psychological care	25 (50.0 %)	12 (52.2 %)	13 (48.1 %)	1.000
Prior psychiatric care	11 (22.0 %)	4 (17.4 %)	7 (25.9 %)	0.515
Use of psychotropic drugs	8 (16.0 %)	1 (4.3 %)	7 (25.9 %)	0.038

EPDS scores were correlated using Pearson's correlation. Raw scores were also included in the multiple linear regression analysis. Due to the small sample size, the authors included in the model only more significant variables. Postnatal diagnosis and use of psychotropic drugs were associated with postpartum depression (Table 3) with an EPDS score of 3.7 and 6.8 for the risk of developing postpartum depression, respectively. There was a trend indicating that gestational diabetes increases the likelihood of puerperal depression.

**Table 3:** Multiple logistic regression variables associated with depression.

**Coefficients**										
Model		Non-standardized coefficients		Standardized coefficients	t	Sig.	95 % confidence interval of B		Collinearity statistics	
		B	Standard error	Beta			Lower limit	Upper limit	Tolerance	VIF
1	(Constant)	4.710	2.632		1.789	.080	−0.588	10.009		
	Postnatal diagnosis	3.748	1.637	.288	2.289	.027	.452	7.044	.958	1.043
	Use of psychotropic drugs	6.819	2.182	.386	3.125	.003	2.427	11.212	.997	1.003
	Gestational diabetes	3.394	1.970	.217	1.723	.092	−0.570	7.359	.959	1.043

Dependent variable: Total.

## Discussion

This original study found that prenatal diagnosis of CCHD was associated with significantly lower levels of depressive symptoms in puerperal women when compared to postnatal diagnosis. Mothers of hospitalized infants often experience high levels of depressive symptoms, but those who have had more time to familiarize themselves with their infant's diagnosis tend to be more optimistic.^14^^,^^15^

This study stresses the importance of fetal echocardiography as part of prenatal screening of pregnant women as prenatal detection of CHD has an impact on the expectant mother's emotional state and may directly affect the mother-baby bond and child development. A comparison of the time of diagnosis of CHD showed that prenatal diagnosis can positively impact the mother's emotional state in the postpartum period.

An infant's diagnosis of CHD is a major complication during pregnancy and a risk to fetal health and is directly associated with postpartum depression. It is thus crucial to clinically diagnose CHD during pregnancy and identify any early depressive symptoms in the expectant mothers.^16^

Prenatal diagnosis can also be a protective factor for depression as expectant mothers may receive specialized emotional care during pregnancy. Given its overall value, fetal echocardiography is an essential part of routine prenatal care.

Prenatal diagnosis is, however, less frequent among families with lower socioeconomic conditions. The present study showed that mothers of infants with the postnatal diagnosis were likely less educated and mostly received care at the public health system. Delayed diagnosis may hinder appropriate care and referral of pregnant women and their children.^17^^,^^18^ Koutra et al. (2018) pointed out that sociodemographic characteristics, including maternal age and low level of education, as well as psychosocial factors, such as depression, are associated with postpartum depression.^19^

The study participants were all puerperal mothers of infants diagnosed with CCHD who were hospitalized. Studies have demonstrated that postpartum depression is a moderate to severe condition usually occurring 4 to 8 weeks after birth and common symptoms include low mood, loss of appetite, altered sleep, fatigue, extreme worry, feelings of guilt and sadness, and low self-esteem.

In the present study, the authors found an association between pre-pregnancy use of psychotropic drugs, such as antidepressants, and postpartum depression, which shows that some mothers had a prior history of emotional distress. Although the authors found a trend indicating an association between gestational diabetes and postpartum depression, gestational diabetes is a common complication of pregnancy and studies have demonstrated that pregnant women with gestational diabetes are at greater risk of developing postpartum depression.^20^. Since the authors have used logistic regression analysis to control the confounder variables, such as age, social and economic condition and quality of prenatal care, we could separate the joint effect of these parameters to assess the specific effect of the outcome: maternal depression after cardiac diagnosis by fetal echocardiography or after birth.

Routine fetal echocardiography in the absence of cardiac risk factors is available in Brazil in the private health system, but not yet in the public system, where only when there is a specific risk factor for heart disease or a suspicion of a cardiac defect at obstetric morphological scan the mother is referred for fetal heart examination, which could account for differences between the two groups.

Postpartum depression is a condition that can affect child development manifesting itself as behavioral and cognitive difficulties.^21^ Children of depressed mothers may develop more negative emotions in their lives, such as anger, guilt, fear and nervousness and interventions aiming at promoting mental health in this population should be implemented.

In addition, the importance of a family-centered care has been emphasized, with recommendation of interventions aimed to decrease parental stress, such as adequate clarification about their babies' conditions and psychological support within hospital units at such a critical moment.^22^^,^^23^

The EPDS was administered to all mothers before surgery, without exceptions, and there have been no refusals.

Among the limitations of this study are the fact that it was carried out in only one site and data collected was temporarily disrupted due to the onset of the COVID pandemic and social isolation requirements. It meant that the authors had to discontinue the study before reaching the required number of participants per group. In addition to these logistical issues, mothers of infants with prenatal diagnosis were likely to be more educated. Thus, education was a confounding factor that may have facilitated access to screening for CHD. Another limitation could be the possibility of prematurity in some babies, considering that mothers of premature neonates might have a trend toward a higher level of depression,^24^ a potential confusion bias to be taken into account in the interpretation of the results.

Future studies with longer follow-ups of child emotional development could be useful to determine if maternal postpartum depression could potentially be related to future emotional and cognitive difficulties in these children.^25^

Prenatal diagnosis of CCHD was associated with significantly lower levels of depressive symptoms in puerperal women when compared to postnatal diagnosis.

## Authors contributions

All authors contributed equally to this work.

## Conflicts of interest

The authors declare no conflicts of interest.
